# Link Prediction on Complex Networks: An Experimental Survey

**DOI:** 10.1007/s41019-022-00188-2

**Published:** 2022-06-21

**Authors:** Haixia Wu, Chunyao Song, Yao Ge, Tingjian Ge

**Affiliations:** 1 College of Computer Science, Tianjin Key Laboratory of Network and Data Security Technology, Nankai University, Tianjin, China; 2 University of Massachusetts Lowell, Massachusetts, United States

**Keywords:** Link prediction, Complex networks, Data mining, Network analysis, 00-01, 99-00

## Abstract

Complex networks have been used widely to model a large number of relationships. The outbreak of COVID-19 has had a huge impact on various complex networks in the real world, for example global trade networks, air transport networks, and even social networks, known as racial equality issues caused by the spread of the epidemic. Link prediction plays an important role in complex network analysis in that it can find missing links or predict the links which will arise in the future in the network by analyzing the existing network structures. Therefore, it is extremely important to study the link prediction problem on complex networks. There are a variety of techniques for link prediction based on the topology of the network and the properties of entities. In this work, a new taxonomy is proposed to divide the link prediction methods into five categories and a comprehensive overview of these methods is provided. The network embedding-based methods, especially graph neural network-based methods, which have attracted increasing attention in recent years, have been creatively investigated as well. Moreover, we analyze thirty-six datasets and divide them into seven types of networks according to their topological features shown in real networks and perform comprehensive experiments on these networks. We further analyze the results of experiments in detail, aiming to discover the most suitable approach for each kind of network.

## Introduction

With the development of network analysis, many complex systems can be described as networks [1]. Networks are a natural and powerful tool for characterizing a large number of social, biological, and information systems composed of interacting elements, and network science is one of the most active interdisciplinary fields of research today. A typical network consists of nodes and edges, where nodes denote various entities in real systems and edges represent the relationships between entities. Treating individuals as nodes for example, and associations between corresponding persons as edges, social relations could be abstracted as a network. Protein–protein interactions form a network where nodes denote proteins and edges denote interactions among them. In addition, the hyperlink structure of the Internet can be modeled as a directed graph. These complex networks have many significant statistical properties, such as the small-world effects and the scale-free properties.

**Related Works** A number of problems related to complex networks are being studied, including community detection and structural network analysis. In recent years, link prediction on complex networks attracts more and more concerns. *Link Prediction* is a fundamental problem that attempts to estimate the likelihood of the existence of a link between two nodes [2], which makes it easier to understand the association between two specific nodes and how the entire network evolves.

The problem of link prediction over complex networks can be categorized into two classes. One is to reveal the missing links. The other is to predict the links that may exist in the future as the network evolves [3]. Previous studies [4–6] suggest that there may be mechanisms to guide the formation of networks; it is therefore important to investigate the evolution of networks, as well as networks’ characteristics and structures.

Link prediction has been widely applied to a variety of fields. In biology, it is used to predict unobserved links in PPI (protein–protein interaction) networks [7–10]. In terms of social networks [11–13], link prediction algorithms help to recommend friends with similar interests or goods that one may purchase [14]. There have been several reviews on link prediction analysis in social networks [15–17]. As for the Internet, researchers use link prediction to realize web page personalization [18].

There are a large number of link prediction methods. Malhi et al [19] give a review on various link prediction algorithms. It focuses on evaluating shortcomings of link prediction methods. However, it does not provide any evaluation results, and the information it provides is rather limited. Lü et al [2] present an excellent survey by summarizing different approaches; introducing typical applications; and outlining future challenges of link prediction algorithms. However, the methods presented in this paper are somewhat antiquated. Martínez et al [20] add to the review of some more recent methods, as well as a more detailed experimental comparison of the similarity-based methods, while the specific data used for the experiments are not analyzed or categorized. As experimentally demonstrated in this survey, it is difficult to give a method that has the best performance in all complex networks, which strongly depends on the structural properties of the network. Therefore, an empirical study of discovering the most suitable link prediction methods for different kinds of networks is desirable. To the best of our knowledge, we are the first to review link prediction methods, including the state-of-the-art network embedding-based methods, on top of a comprehensive evaluation result.

**Contributions** The evaluation comparison of the most advanced network embedding-based link prediction methods is included in this paper, as well as other popular traditional methods. We also summarize and analyze the trade-offs among different methods. This work has greatly compensated for the shortcomings of previous research articles. In this work, we divide the complex networks involved in some common applications into seven categories and analyze their characteristics by calculating their attributes. The structural features of different kinds of networks are also extracted. On the basis of comprehensive experiments, we recommend appropriate link prediction methods for each type of networks.

In this study, we focus on the link prediction problem on undirected networks which can be formulated as follows. Consider an undirected network *G*(*V*, *E*), where *V* represents a set of nodes and *E* stands for a set of edges. Using *U* to denote the set of all possible links, the target of link prediction is to infer the missing links or links that will arise in the future in $U- E$. Our contributions are summarized as follows:

- A rational categorizing for link prediction methods is suggested, and a thorough study of the representative link prediction approaches and methods, including the state-of-the-art network embedding-based methods, is performed. Due to the emergence of the large number of the network embedding (graph representation learning)-based methods in recent years, we are not able to make a comprehensive summary of them all. Instead, we selected several representative methods for investigation, reflecting the commonness of this kind of methods. The characteristics of these methods are summarized and compared (Sect. 2)
- We present the properties used to classify complex networks and introduce the characteristics of each type. A new taxonomy of complex networks is then proposed (Sect. 3)
- To the best of our knowledge, this survey is the first comprehensive evaluation of a broad spectrum of link prediction methods and includes the evaluation comparison of the state-of-the-art network embedding methods. A mass of real datasets are comprehensively tested to compare a large number of link prediction methods. A rounded analysis is conducted according to the experimental results for each type of networks, which is able to give instructional selection advice for different link prediction tasks (Sect. 4)

**Fig. 1:**
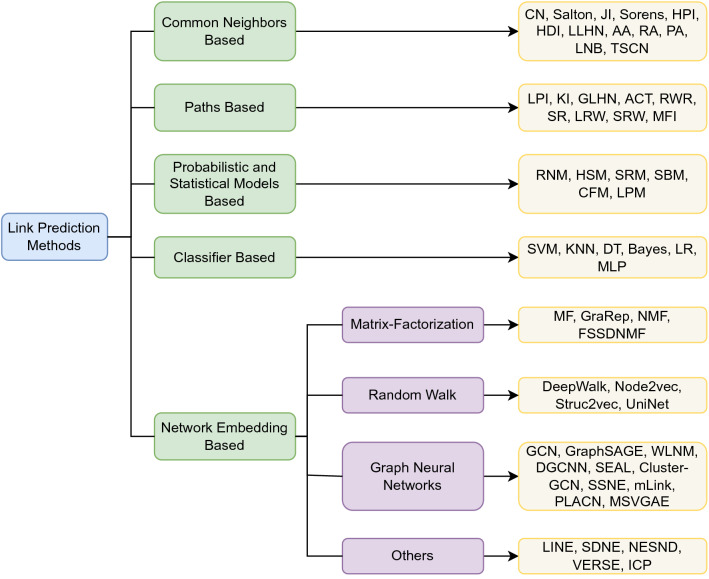
Taxonomy for link prediction methods

**Fig. 2:**
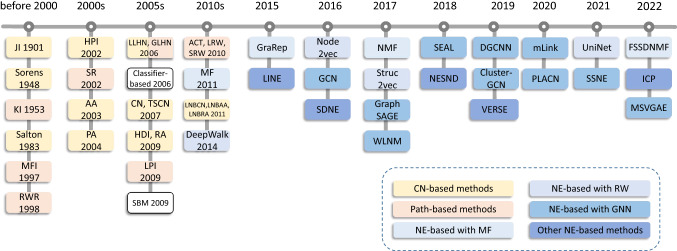
Timeline of link prediction methods

## Methods for Link Prediction

Researchers have proposed a variety of link prediction techniques, ranging from the simplest heuristic methods of counting common neighbors between two nodes to the current popular network embedding-based methods. Most of them calculate the similarities or the probabilities of forming links between nodes by capturing the structural features of the network. In this section, we perform a comprehensive overview of representative link prediction approaches and propose a new taxonomy for link prediction methods (as shown in Fig. 1), including common neighbor-based, path-based, probabilistic and statistical models-based, classifier-based, and network embedding-based methods. In Sect. 2.6, a more detailed comparison among different methods are given, including time complexity and scalability, etc. Table 1 explains the meaning of the common notations that will be used in this survey.

The timeline for the development of link prediction methods is organized in Fig. 2. As can be seen from the figure, before 2010, the traditional link prediction methods were the mainstream methods, such as common neighbor-based and path-based methods, which were widely applied because of their simplicity, interpretability, high efficiency, and high accuracy. However, these methods fail to make full use of nodes and network structure information. With the rapid development of Internet technology and big data, the scale of the network continues to expand. The traditional adjacency matrix $A\in R^{N\times N}$ representing graph structure information presents high-dimensional and sparse characteristics, which poses a challenge to the research on large-scale networks. Probabilistic and statistical-based methods are time-consuming and computationally expensive, making them unsuitable for large-scale networks. Classifier-based methods [21] face class imbalance due to the sparsity of real networks, that is, the number of nonexistent links between nodes far exceeds the number of existing links. The network embedding methods, also known as graph representation learning, effectively address the deficiencies of the traditional methods. Using the network embedding methods with powerful representation ability, on the premise of retaining the network structure information, the nodes are mapped into the low-dimensional space, and the low-dimensional and dense continuous feature vector representation of each node is obtained. DeepWalk [22] is the first method to use deep learning for network embedding. It obtains a linear sequence of network structure through random walk and further uses the SkipGram model in word representation learning to learn the representation of nodes in the network. On the basis of DeepWalk, after 2015, with the development of graph representation learning, more and more network embedding methods have been applied to link prediction tasks. As a representative class of methods, the graph neural network methods are extremely effective methods to solve the problem of graph learning by adding graph operations to the traditional deep learning model and applying the structural information and attribute information of the graph to deal with the complexity of graph data.

**Table 1:** A summary of common notations

*G*(*V*, *E*)	Undirected network
*V*	Set of nodes
*E*	Set of edges
*n*	Number of nodes
*m*	Number of edges
$s_{xy}$	Similarity score of node *x* and node *y*
$d_m$	Maximum degree of a network
$d_min$	Minimal degree of a network
*d*(*v*)	Number of edges connecting to node *v*
$\Gamma _x$	Set of neighbors of node *x*
*l*	Number of random walk steps
*L*	Laplacian matrix
*S*	Similarity matrix
*M*	Direct similarity
*A*	Adjacent matrix
*I*	Identity matrix
$a, \epsilon , \beta , \phi , \psi$	Parameters
*P*	Transition probability matrix
$\pi _{xy}$	Probability of a walker starting from *x* and locating at *y*

### Methods Based on Common Neighbor

Common neighbor(CN)-based methods assign a score $s_{xy}$ for each pair of nodes *x* and *y*, which is proportional to the probability that there exists an edge between *x* and *y*. It is an apparent intuition that two nodes *x* and *y* are more likely to form a link in the future, if their neighbors have large overlap. The simplest technique of measuring common neighbor is counting the shared neighbors directly which is called *Common Neighbors* (CN). As a basis of research work presented later, it is also applied to the study of graph streams [23] and dynamic social networks [24]. It can be computed as Equation (1). For a node *x*, let $\Gamma (x)$ denote the neighbors of *x* in *G*(*V*, *E*).

Other representative methods of calculating $s_{xy}$ based on common neighbor are *Salton Index* (Salton) [25], *Jaccard Index* (JI) [26], *Sørensen Index* (Sørensen) [27], *Hub Promoted Index* (HPI) [28], *Hub Depressed Index* (HDI) [29], *Local Leicht–Holme–Newman* (LLHN) [30], *Adar-Adamic Index* (AA) [13], *Resource Allocation* (RA) [29], *Preferential Attachment* (PA) [31]. In summary, these metrics are variations based on the CN method, which are normalized or take into account the importance of neighbors in order to minimize biases due to node degree skewness. They are calculated as follows.

$$\begin{aligned} \begin{array}{lllllllll} \mathrm {s}_{x y}^{C N}=\left| \Gamma _{x} \cap \Gamma _{y}\right| &{} (1)&{}&{} \quad \mathrm {s}_{x y}^{Salton}=\frac{\left| \Gamma _{x} \cap \Gamma _{y}\right| }{\sqrt{\left| \Gamma _{x}\right| \left| \Gamma _{y}\right| }} &{}(2)\\ s_{x y}^{J I}=\frac{\left| \Gamma _{x} \cap \Gamma _{y}\right| }{\left| \Gamma _{x} \cup \Gamma _{y}\right| } &{} (3) &{}&{} \quad s_{x y}^{\text{ Sorensen } }=\frac{2\left| \Gamma _{x} \cap \Gamma _{y}\right| }{\left| \Gamma _{x}\right| +\left| \Gamma _{y}\right| } &{} (4) \\ s_{x y}^{H P I}=\frac{\left| \Gamma _{x} \cap \Gamma _{y}\right| }{\min \left\{ \left| \Gamma _{x}\right| ,\left| \Gamma _{y}\right| \right\} } &{}(5) &{}&{} \quad s_{x y}^{H D I}=\frac{\left| \Gamma _{x} \cap \Gamma _{y}\right| }{\max \left\{ \left| \Gamma _{x}\right| , \left| \Gamma _{y}\right| \right\} }&{}(6) \\ s_{x y}^{L L H N}=\frac{\left| \Gamma _{x} \cap \Gamma _{y}\right| }{\left| \Gamma _{x}\right| \left| \Gamma _{y}\right| } &{} (7) &{}&{} \quad \mathrm {s}_{x y}^{A A}=\sum _{w \in \Gamma _{x} \cap \Gamma _{y}} \frac{1}{\log \left| \Gamma _{w}\right| }&{}(8)\\ s_{x y}^{R A}=\sum _{w \in \Gamma _{x} \cap \Gamma _{y}} \frac{1}{\left| \Gamma _{w}\right| }&{}(9) &{}&{} \quad s_{x y}^{P A}=\left| \Gamma _{x} \Vert \Gamma _{y}\right| &{}(10)\\ \end{array} \end{aligned}$$

$\bullet$ **Local Naive Bayes (LNB) **[32] It is a method based on the Bayesian theory, while combining the idea that different shared neighbors play different roles. The formula of the connection likelihood is

11 $$\begin{aligned} s_{xy}^{LNB}= \sum _{w \in \Gamma _x \cap \Gamma _y}^{} {f(|\Gamma _w|)log(aR_w)}, \end{aligned}$$

where *f* has three forms, which are $f(|\Gamma _w|) = 1$, $f(|\Gamma _w|) = \frac{1}{\log |\Gamma _w|}$, and $f(|\Gamma _w|) = \frac{1}{|\Gamma _w|}$, corresponding to the CN, AA and RA measurements, respectively. In Equation (11), *a* is a constant for a given training set and $R_w$ is the role function of the node *w*, which can be defined as in [20]:

12 $$\begin{aligned} R_w = \frac{|{e_{x,y}: w \in \Gamma _x \cap \Gamma _y, e_{x,y} \in E}| + 1}{|{e_{x,y}: w \in \Gamma _x \cap \Gamma _y, e_{x,y} \notin E}| + 1}. \end{aligned}$$

$\bullet$ **Transfer Similarity (TS) **[33] Direct similarities are less accurate when a network is sparse. Thus, transfer similarity that properly integrates the high-order correlations is proposed [34]. The self-consistent definition of this index is

13 $$\begin{aligned} S = \epsilon MS + M, \end{aligned}$$

where *M* represents the direct similarity, such as common neighbor (TSCN) or Pearson correlation coefficient, and $\epsilon$ is the rate of information aging when the information is further transferred.

### Methods Based on Path

The common neighbor-based approaches ignore the global similarities between nodes and can only capture limited local structural information. In contrast, the path-based methods formulate similarity measurements according to the paths between nodes and take care of more high-order information, which greatly alleviate the previous problem. We let $s_{xy}$ measure the possibility of the appearance of a link between *x* and *y* which has the same meaning as in Sect. 2.1. In this subsection, *A*, *I*, and *S* represent the adjacent matrix, identity matrix, and similarity matrix of *G*(*V*, *E*), respectively.

$\bullet$ **Katz Index (KI) **[35] Katz index is defined as

14 $$\begin{aligned} s_{xy}^{KI}=\sum _{l=1}^{\infty }\beta ^l \centerdot |paths_{xy}^{<l>}| = \sum _{l=1}^{\infty }\beta ^l(A^l)_{xy}, \end{aligned}$$

where $|paths_{xy}^{<l>}|$ is the number of the *l*-length paths between nodes *x* and *y*, and $\beta$ is a damping factor used to control the attenuation pace ($0 \le \beta \le 1$). The Katz index for all pairs of nodes can be computed by

15 $$\begin{aligned} S = (I - \beta A)^{-1} - I. \end{aligned}$$

$\bullet$ **Local Path Index (LPI) **[3] This index takes local paths into consideration [20]. It reduces the complexity of Katz index at the cost of accuracy by only focusing on the paths whose length are 2 or 3, which can be defined as

16 $$\begin{aligned} S = A^2+\epsilon A^3, \end{aligned}$$

where $\epsilon$ is a free parameter like $\beta$.

$\bullet$ **Global Leicht–Holme–Newman (GLHN) **[30] The definition of this index consists of two parts: the neighbor term, and the self similarity. The initial guess is

17 $$\begin{aligned} s_{xy}^{GLHN}=\phi \sum _{w}^{}A_{iw}S_{wj}+\psi \delta _{ij}, \end{aligned}$$

where $\delta _{ij}$ is the Korenecker’s function [36], while $\phi$ and $\psi$ are free parameters that control the balance of the two parts.

$\bullet$ **Local Random Walk (LRW) **[37] Random walk is a process that a walker starts from a source and chooses one of the neighbors randomly as his next step [11]. It can be described by a Markov chain and its transition probability matrix. We use *P* to denote the transition probability matrix, and $\pi _{xy}(l)$ to denote the probability that a walker starts from node *x* and reaches the node *y* after *l* steps [37]; thus We have

18 $$\begin{aligned} \overrightarrow{\pi _x}(l)=P^T\overrightarrow{\pi _x}(l-1), \end{aligned}$$

where $\overrightarrow{\pi _x}(0)$ is a vector of length |*V*| with the *x*-th element equals to 1 and others to 0.

The similarity is calculated as

19 $$\begin{aligned} s_{xy}^{LRW}(l) = \frac{|\Gamma _x|}{2|E|}\pi _{xy}(l) + \frac{|\Gamma _y|}{2|E|}\pi _{yx}(l). \end{aligned}$$

It reduces the computational cost by limiting the random walk steps *l*. A shortcoming of this metric is its sensitivity to the regions far away from the target [11].

$\bullet$ **Superposed Random Walk (SRW) **[37] To counteract the dependency of local random walk, Liu et al proposed to continuously release the walkers at the source. By superposing the contribution of each walker, the similarity index is

20 $$\begin{aligned} s_{xy}^{SRW}(l)= \sum _{l=1}^{t}s_{xy}^{LRW}(l), \end{aligned}$$

$\bullet$ **Random Walk with Restart (RWR) **[38] Staring from a node in *G*, each step has two choices: return to the source node with probability $\alpha$ or go to its neighbors randomly with probability $1 - \alpha$. There is an iterative equation:

21 $$\begin{aligned} \overrightarrow{\pi _x} = \alpha P^T \overrightarrow{\pi _x}+(1-\alpha )\overrightarrow{e_x}, \end{aligned}$$

where $\overrightarrow{\pi _x}$ is a vector whose term is the probability of the walker locating at the corresponding node when the walking process reaches a steady state, while $\overrightarrow{e_x}$ is a vector of length *n* with the *x*-th element equals to 1 and others to 0. Finally, use $\pi _{xy}$ denotes the probability of a random walker starting from *x* and locating at *y* in the steady state, and the random walk with restart similarity is defined as

22 $$\begin{aligned} s_{xy}^{RWR} = \pi _{xy}+\pi _{yx}. \end{aligned}$$

$\bullet$ **Average Commute Time (ACT) **[37] The average commute time between *x* and *y* is the sum of the average steps from *x* to *y*, and from *y* to *x*, which can be computed by the pseudoinverse of the Laplacian matrix $L^+$. Therefore, the average commute time can be expressed as

23 $$\begin{aligned} s_{xy}^{ACT} = \frac{1}{L_{xx}^+ + L_{yy}^+ -2L_{xy}^+}. \end{aligned}$$

$\bullet$ **SimRank (SR) **[39] Suppose two random walkers start from *x* and *y*, respectively, this index reflects the time that they are expected to meet. A recursive equation for $s_{xy}$ is

24 $$\begin{aligned} s_{xy}^{SR} = \frac{C}{|\Gamma _x||\Gamma _y|}\sum _{u=1}^{|\Gamma _x|}\sum _{w=1}^{|\Gamma _y|}s(\Gamma _u(x),\Gamma _w(y)), \end{aligned}$$

where C is a constant between 0 and 1.

$\bullet$ **Others** *Matrix Forest Index* (MFI) [40]: This index is also a method of calculating similarities and is proposed based on matrix-forest theorem which can be written as

25 $$\begin{aligned} S = (I + L)^{-1}. \end{aligned}$$

### Methods Based on Probabilistic and Statistical Models

Probabilistic and statistical methods provide a way to extract the underlying structure from a network. They build a model and estimate the model parameters which can best fit the data of the network, and then predict the formation probability of the missing links. These methods are highly time-consuming for model training, so they are impractical for large networks. Moreover, they only have mediocre prediction results. On the other hand, they do provide valuable insights into the network structure. Based on the above considerations, we only conduct experiments on the stochastic block model (SBM) as a representative.

**Stochastic Block Model (SBM) **[41]: In a stochastic block model, nodes are divided into different groups and the probability that two nodes are connected relies only on the groups which they belong to. This model is based on three properties: Nodes in real networks (1) are usually organized in communities, (2) play distinct roles, and (3) connect to each other based on these rules. The probability that a link truly exists requires to calculate all possible partitions of the network. Thus, Metropolis sampling algorithm [42] can be used to correctly sample relevant partitions and obtain an estimation of the link probability in practice. When the number of possible partitions is very large, this approach is computationally expensive.

**Others** Here is a brief introduction of other selective probabilistic and statistical-based methods. *Relational network model* (RNM) [17, 43] is originally designed for attribute prediction over a database. Due to the difference of trained models, RNM can be divided into *Relational Bayesian Networks* (RBN) [44], *Relational Markov Networks* (RMN) [45] and *Relational Dependency Networks* (RDN) [46]. *Hierarchical structure model* (HSM) [47] is suitable for networks which exhibit hierarchical organizations such as metabolic networks. In *Stochastic Relational Model* (SRM) [48], the relationships between nodes are modeled by a tensor interaction of multiple Gaussian processes. Huang [49] proposes a framework of predicting links, *cycle formation model* (CFM), based on the cycle formation which relates to the generalized clustering coefficient measure. *Local probabilistic model* (LPM) [50] learns a local Markov random field model constrained on non-derivable frequent itemsets from the local neighborhood and forms the co-occurrence probability feature.

### Methods Based on Classifier

Link prediction can be studied as a supervised or semi-supervised learning task. A plethora of classification algorithms are applicable for link prediction [21]. Choosing appropriate features is the most critical part of a supervised learning algorithm. Due to the large number of classification methods, we choose six representative classifiers for evaluation, including *Support Vector Machine* (SVM) [51], *K-Nearest Neighbors* (KNN) [52], *Decision Tree* (DT) [53], *Naive Bayes* (Bayes) [54], *Logistic Regression* (LR) [55], and *Multilayer Perceptron* (MLP) [56], where the training features include the indices mentioned in Sects. 2.1 and 2.2 . The indices with a high time complexity are not considered, such as TS, GLHN, SRW, RWR, ACT, SR, and MFI. Other classifier based methods are introduced as follows.

Hasan et al [21] choose proximity features, aggregated features, and topological features. Lichtenwalter et al [57] provide a general, high-performance supervised framework for the prediction task, and try to overcome the imbalance by oversampling and undersampling. De Sá et al [58] use the metrics computed from the network structure, and the weights of links are taken into consideration. In addition, Doppa et al [59] propose a learning algorithm based on the chance constrained programs which exhibit all the properties needed for a good link predictor. The idea of Chen et al [60] is to reduce the computation cost by combining multiple classifiers while maintaining the accuracy of predictions.

Kashima et al [61] propose a semi-supervised link prediction method called Link Propagation by applying the label propagation technique, where the Kronecker sum similarity is used as the similarity matrix. However, the time complexity and the space complexity makes it unrealistic to deal with large networks. Raymond et al [62] extend the semi-supervised learning algorithm [61] to solve the link prediction problem approximately on large-scale dynamic graphs by using a non-trivial combination of techniques in the linear algebra. Moreover, Zeng et al [63] give a new semi-supervised learning approach SLiPT. The entire algorithm is based on the temporal features.

### Methods Based on Network Embedding

The emergence of large-scale complex networks has led to dimensionality explosion, so network embedding(NE)-based methods are needed to reduce the dimensionality, and capture the charactersitcis and attributes of the network at the same time; therefore they can be applied to link prediction. Different from the traditional adjacency matrix, network embedding aims to effectively preserve rich topological and structural information such as links, neighbors, and high-order proximities [64, 65] by embedding nodes into a low-dimensional space to predict the possible future links. The previous high-dimensional sparse feature vectors can be represented by the low-dimensional dense embedding vectors.

A good network embedding method should be able to capture the internal structure of the network well to predict the possible future links. We divide network embedding methods into shallow and deep network embedding techniques according to their different encoding methods. It can also be subdivided into matrix factorization based, random walk based, graph neural network based, and other methods.

#### Network Embedding with Matrix Factorization

The traditional algorithms of network embedding consider the problem of network embedding as matrix decomposition or matrix dimensionality reduction, and reduce the dimensionality of the adjacency matrix of the graph by matrix decomposition or singular value decomposition, so that the original network structure can be easily restored by learning the embedding vectors. Matrix factorization-based network embedding is widely applied to recommender systems [66]. It represents the attributes of the network (such as the similarities of node pairs) in the form of a matrix, which is factored to obtain node embeddings. Inspired by traditional dimensionality reduction techniques, network embedding can be regarded as a dimensionality reduction problem with retained structure.

$\bullet$ **MF **[67] Menon and Elkan propose a latent feature learning method which extends matrix factorization to solve structural link prediction problems in graphs. It extracts the latent features of nodes and use them for prediction tasks. The similarity matrix *S* is factorized to

26 $$\begin{aligned} S \thickapprox L(U\Lambda U^T), \end{aligned}$$

where we have $U \in {\mathbb {R}}^{n \times k}$, $\Lambda \in {\mathbb {R}}^{k \times k}$, and $L(\cdot )$ is a link function. Each node *x* will have a latent vector $u_x \in {\mathbb {R}}^k$, where *k* is the number of latent features [67, 68]. The similarity is calculated as

27 $$\hat{S}_{{xy}} (U,\Lambda ) = L(u_{i}^{T} \Lambda u_{j} ).$$

$\bullet$ **GraRep **[69] It considers the *k* order (*k*>2) similarity. Although GraRep can get the node representation with stronger expression ability, it takes a lot of time to calculate the power of a matrix and SVD. GraRep similarly exploits node co-occurrence information at different scales by raising the graph adjacency matrix to different powers. Singular value decomposition (SVD) is applied to the powers of the adjacency matrix to obtain a low-dimensional representation of nodes.

$\bullet$ **FSSDNMF **[70] To address the network noise problem, a novel link prediction model based on deep nonnegative matrix factorization is proposed, which elegantly fuses topological and sparse constraints to perform the link prediction task. The observed link information of each hidden layer is fully exploited by deep nonnegative matrix factorization. The similarity score is then calculated and mapped to a multilayer low-dimensional latent space using the common neighbor method to obtain topological information for each hidden layer. At the same time, a norm-constrained factor matrix is used at each hidden layer to remove random noise.

In practical applications, nonnegative matrix factorization (NMF) and singular value decomposition (SVD) are usually used to get the approximation of *S*, whose time complexity is $O(n^3)$. Duan et al [71] applied the structural bagging to decompose the link prediction problem into smaller pieces, and use NMF to factorize the adjacency matrix, which addresses the top-k problem in link prediction.

#### Network Embedding with Random Walk

Only decomposing the adjacency matrix can only take into account the influence of the direct neighbor on the current node, which is very limited. Random walk is used to generate the context of nodes which makes up for the deficiency of matrix factorization. Then the node sequences can be treated as sentences to take advantage of natural language processing methods to get node embeddings. Under this circumstance, the more times two nodes appear in the same random walk, the more similar their embeddings will be.

$\bullet$ **DeepWalk **[22] This method is the pioneering work to learn nodes’ vector representations using random walks which obtains local information by truncated random walks to generate the context of nodes and thereby learns latent representations by treating node sequences as sentences. It provides a new idea for network embedding algorithms, which is often used as a benchmark model for this kind of method. By performing random walks on the network, the node sequence is obtained, and the vector representation of the node is learned by using the skip-gram model in natural language processing.

$\bullet$ **Node2vec **[72] Grover et al proposed Node2vec, which learns continuous feature representations of nodes. It further utilizes a biased random walk strategy that combines breadth-first search(BFS) and depth-first search(DFS) neighborhood exploration to capture a more flexible contextual structure on the basis of DeepWalk. Nodes that are “close” in the network will tend to be “close” in the latent representation space.

$\bullet$ **Struc2vec **[73] Struc2vec pays attention to the structural identity and uses a hierarchical metric to measure node similarity at different scales by constructing a weighted multilayer graph to generate context. It defines vertex similarity from the perspective of spatial structural similarity.

$\bullet$ **UniNet **[74] The existing network embedding models based on random walk are unified into an optimized framework which can be effectively used for large-scale network. The Metropolis-Hastings sampling is adopted for edge sampling, which greatly improves the efficiency of random walk generation in network representation learning model.

However, the above approaches merely provide the embedding vectors for subsequent analysis tasks, and we still need to apply similarity calculation and so on for link prediction. For example, Euclidean distance, standardized Euclidean distance, Chebyshev distance, and cosine distance can be used to compute the similarities. In a previous set of experiments, we have evaluated the results of using different distance metrics in different network embedding methods for link prediction. The results did not reflect a significant influence among different distance metrics. Since cosine similarity is the most commonly used metric in network embedding literature, we apply cosine distance between two nodes to quantify their similarities in this work as well.

#### Network Embedding with Graph Neural Networks

Graph neural networks (GNNs) are proposed based on convolutional neural networks (CNNs) and graph embedding. Firstly, traditional CNNs can only operate on regular Euclidean space-based data such as images and text, while complex networks are non-Euclidean data structures. Secondly, although shallow encoding methods such as DeepWalk and Struc2vec have achieved breakthroughs in graph embedding, many of them still suffer from their shallow learning mechanisms, the network embedding quality can hardly be further improved. Thus GNNs are brought forward to solve the above problems [75]. There are three most popular downstream graph analysis tasks, namely node classification, graph classification, and link prediction. While there is abundant literature on the first two, GNNs for link prediction is relatively less studied and less understood. The following lists some representative methods of them.

$\bullet$ **Graph Convolutional Networks(GCN) **[76] This model is based on an efficient variant of CNNs for semi-superivised learning on graph data. It learns hidden layer representations that encode both local graph structure and features of nodes, so that we can use these characteristics to complete the tasks such as node classification, graph classification, and link prediction.

$\bullet$ **GraphSAGE **[77] It is an inductive learning framework that can efficiently generate the unknown vertex embedding vectors by learning a function that aggregates the neighbor vertices.

$\bullet$ **WLNM **[78] This is a new link prediction framework proposed to automatically learn network topology features. The framework first extracts a enclosing subgraph for each target link, and then encodes the subgraph into an adjacency matrix. Finally, the neural network is trained on these adjacency matrices and the prediction model is learned. A fast hashing-based Weisfeiler-Lehman (WL) algorithm is proposed to mark vertices according to their structural roles in subgraph while preserving the inherent directionality of the subgraph.

$\bullet$ **DGCNN **[79] Zhang et al proposed a novel end-to-end deep learning architecture for graph classification, called Deep Graph Convolutional Neural Network. Since features can be extracted using a novel spatial graph convolution layer, it also can be used for link prediction. It learns from the topology of the global graph by sorting vertex features rather than adding them together, which is supported by the new SortPooling layer.

$\bullet$ **SEAL **[80] SEAL extracts local subgraphs that preserve rich information and learns heuristics suitable for the current graph by a GNN. It will obtain a function that takes local enclosing subgraphs as input and outputs the possibility that the links exist. SEAL is flexible with which GNN or node embeddings to use. We follow the default setting of original paper, that is, choose DGCNN as the default GNN and select Node2vec as the default embeddings.

$\bullet$ **Cluster-GCN **[81] It is an efficient algorithm for training deep and large GCN. Cluster-GCN works as the following: at each step, it samples a block of nodes that associate with a dense subgraph identified by a graph clustering algorithm, and restricts the neighborhood search within this subgraph. This simple but effective strategy has made significantly improvement on memory and computational efficiency, while being able to achieve comparable test accuracy with previous algorithms.

$\bullet$ **Others** [82] introduces Attention Mechanisms into Graph Neural Networks which is called GAT. Each layer learns the contribution of each neighbor of the node to its new feature generation, and aggregates the neighbor features according to the contribution degree to generate new aggregated features for downstream tasks. Cai et al [83] introduce a new method for node aggregation, mLink, which can transform the enclosing subgraph to different scales while preserving the network structure information, thus providing supplementary information for link prediction. In order to solve low accuracy on some networks, [84] proposed a method of extracting subgraph for target link based on common neighbors on the basis of WLNM and SEAL, which is called PLACN. After labeling the extracted subgraphs based on the average hop number and average weight, the feature matrix is constructed and finally the convolutional neural network is trained. Guo et al [85] proposes a novel graph embedding framework, called Multiscale Variational Graph Autoencoder (MSVGAE), which learns multiple sets of low-dimensional vectors of different dimensions to represent the mixed probability distribution of the original graph data by the graph encoder. Perform multiple sampling on each dimension. In addition, a self-supervised learning strategy (ie, graph feature reconstruction-assisted learning) is introduced to make full use of graph attribute information to help graph structure learning.

GNNs have become powerful tools for learning over graph-structured data since they showed up, and have been successfully used in link prediction as well. A large number of experiments show that GNN-based methods can learn more effective link representations than previous methods.

#### Other Methods

We present other representative network embedding-based methods which can hardly be divided into any of the previous categories in the last subsection.

$\bullet$ **LINE **[86] This method learns a *d*-dimensional feature representations in two separate phases. In the first phase, it learns *d*/2 dimensions by BFS-style simulations over immediate neighbors of nodes. In the second phase, it learns the next *d*/2 dimensions by sampling nodes strictly at a 2-hop distance from the source nodes. Additionally, it adopts negative sampling [87] to optimize the skip-gram model, compared with the hierarchical softmax [88] used in DeepWalk.

$\bullet$ **SDNE **[89] This algorithm extends the traditional deep autoencoder to preserve the proximity between 2-hop neighbors. It is the first method to introduce the deep learning model into the network representation learning which optimizes first-order and second-order similarity simultaneously. It learns node representations using semi-supervised learning. On the one hand, supervised learning is used to get the local structure from the adjacency matrix to achieve the first-order similarity. On the other hand, unsupervised learning is used to obtain the global structure to meet the second-order similarity. In this way, SDNE can preserve the highly-nonlinear local-global network structure well and address sparsity problems.

$\bullet$ **NESND **[90] It compares the structural similarity algorithm and the network embedding algorithm. On this basis, Cao et al present a new method to supplement local structure information with network embedding algorithm. While this method is only a combinatorial optimization of the existing methods, its characteristics are not listed separately.

$\bullet$ **VERSE **[91] Tsitsulin et al propose a scalable algorithm for graph embeddings, which is extremely efficient and can reach linear time complexity. It falls in between deep learning approaches and the direct decomposition of the similarity matrix. It explicitly learns the distribution of any chosen vertex similarity measure for each graph vertex by training an expressive single-layer neural network.

**Table 2:** A summary of methods

Category	Method	Preserved proximity	Time complexity	S	Learning model
Common neighbor based	CN [92], Salton [25], JI [26], Sorens [27], HPI [28], HDI [29], LLHN [30], PA [31], LNBCN [32]	$1_{st}$ order	$O(d_{m}^2n)$ $\sim$ $O(d_{m}^3n)$	$\checkmark$	Unsupervised
	AA [13], RA [29], LNBAA [32], LNBRA [32]	$2_{nd}$ order	$O(d_{m}^3n)$	$\checkmark$	Unsupervised
	TSCN [33]	$k_{th}$ order	$O(n^3)$	$\times$	Unsupervised
Path Based	LPI [3]	$2_{nd}$ $\sim$ $3_{rd}$ order	$O(d_{m}n^2)$	$\checkmark$	Unsupervised
	KI [35], GLHN [30], ACT [37], RWR [38], SR [39], MFI [40]	$k_{th}$ order	$O(n^3)$	$\times$	Unsupervised
	LRW [37], SRW [37]	$l_{th}$ order	$O(ld_{m}n^2)$	$\checkmark$	Unsupervised
Probabilistic and statistical models based	SBM [41]	$k_{th}$ order	–	$\times$	Supervised
Classifier based	SVM [51], KNN [52], DT [53], Bayes [54], LR [55], MLP [56]	$1_{st}$ $\sim$ $2_{nd}$ order	$O(d_{m}^3n)$ $\sim$ $O(n^2)$	$\checkmark$	Supervised
Network embedding based	MF [67]	$1_{st}$ $\sim$ $2_{nd}$ order	$O(n^3)$	$\checkmark$	Supervised
	GraRep [69]	$2_{nd}$ $\sim$ $k_{th}$ order	$O(mn+d_in^2)$	$\checkmark$	Supervised
	DeepWalk [22]	$2_{nd}$ $\sim$ $k_{th}$ order	$O(d_{i}n\log n)$	$\checkmark$	Unsupervised
	Node2vec [72]	$2_{nd}$ $\sim$ $k_{th}$ order	$O(d_{i}rn)$	$\checkmark$	Semi-supervised
	Struc2vec [73]	Structural Identity	$O(n^3)$	$\checkmark$	Unsupervised
	UniNet [74]	$1_{st}$ $\sim$ $k_{th}$ order	–	$\checkmark$	Semi-supervised
	GCN [76]	$1_{st}$ $\sim$ $k_{th}$ order	$O(d_im+d_i^2n)$	$\times$	Semi-supervised
	GraphSAGE [77]	$1_{st}$ $\sim$ $k_{th}$ order	$O(d_i^2r^{L_n}n)$	$\checkmark$	Unsupervised
	WLNM [78]	$1_{st}$ $\sim$ $k_{th}$ order	–	$\checkmark$	Supervised
	DGCNN [79]	$1_{st}$ $\sim$ $k_{th}$ order	–	$\checkmark$	Semi-supervised
	SEAL [80]	$1_{st}$ $\sim$ $2_{nd}$ order	–	$\checkmark$	Semi-supervised
	Cluster-GCN [81]	$1_{st}$ $\sim$ $k_{th}$ order	$O(d_im+d_i^2n)$	$\checkmark$	Semi-supervised
	LINE [86]	$1_{st}$ $\sim$ $2_{nd}$ order	$O(d_{i}m)$	$\checkmark$	Supervised
	SDNE [89]	$1_{st}$ $\sim$ $2_{nd}$ order	*O*(*mn*)	$\checkmark$	Semi-supervised
	VERSE [91]	$1_{st}$ $\sim$ $2_{nd}$ order	$O(d_{i}rn)$	$\checkmark$	Semi-supervised

Let $d_{m}$ denotes the maximum degree of a network, l denotes the number of the random walk steps. For embedding approaches, $d_{i}$ denotes the dimensionality of embedding vector, $L_n$ is number of layers, r is the number of sampled neighbors per node

$\bullet$ **ICP **[93] A novel link prediction method ICP based on inductive matrix completion is proposed, which recovers the node connection probability matrix by applying node features to a low-rank matrix. The method first explores comprehensive node feature representations by combining different structural topology information with node importance attributes through feature construction and selection. The selected node features are then used as input for a supervised learning task of solving low-rank matrices. The node connection probability matrix is finally recovered by a bilinear function that predicts the connection probability between two nodes and its features and a low-rank matrix.

### Summary

In this section, a new taxonomy is proposed to scientifically divide link prediction methods into five categories. As far as we know, there has been no experimental survey of network embedding-based link prediction methods, especially GNN based, which have currently widely been used for a variety of tasks. In order to address this problem, we have carried out an extensive experimental study on network embedding methods, which are refined to matrix decomposition based, random walk based, graph neural network based, etc. Table 2 provides a clear comparison among the methods from multiple perspectives and offers instructive suggestions for method selection by summarizing the common characteristics of different methods. It can be learned whether the method captures local or global topology information from the aspect of preserved proximity. The time complexities of the link prediction methods mentioned in this section are shown in the fourth column, where “-” indicates that there is no clear time complexity to refer to. The *S* column stands for the scalability of a method, which is limited by the memory requirements and time costs needed for training. The last column represents the learning models of the methods.

## Complex Networks

Complex networks have been used widely to model a large number of relationships. A typical network consists of nodes and edges, where nodes denote various entities in real systems and edges represent the relationships between entities. In this study, we focus on the link prediction problem on undirected homogeneous networks. That is, there is no difference between the edge from *u* to *v* and the edge from *v* to *u*; both are the edge *u*, *v*. Consider a simple network *G*(*V*, *E*), where *V* and *E* are collections of nodes and links, respectively, the directionality and weight of links are ignored, and multiple links and self-connections are not allowed. By observing many properties of actual networks and combining them with link prediction application areas, we roughly categorize the well-known applications into seven kinds of complex networks according to their natural meanings: coauthorship networks, computer networks, infrastructure networks, interaction networks involving people, protein–protein interaction networks, offline social networks, and online social networks.

### Properties

As stated by Newman [94], many studies have proposed some topological features where different types of networks may share a different set of common features. We describe six properties in this paper to distinguish different types of networks. We are mainly concerned with representative features and examine their relationship with link prediction. Common notations are listed in Table 1. We next describe the six properties as following:

$\bullet$ **Average Degree (AD)** Node degree is a basic feature which reflects local information of a node by counting the number of links connected to the node. Average degree is the average of all nodes’ degrees , which measures the overall connectivity of a network and characterizes the intensiveness of connections between nodes. It is defined as

28 $$\begin{aligned} AD = \frac{1}{n}\sum _{v \in V}^{}d(v). \end{aligned}$$

Networks with higher AD usually have higher cohesion and therefore algorithms that can capture local information are more advantageous in such networks.

$\bullet$ **Clustering Coefficient (CC)** Clustering coefficient is a main index to measure clustering numerically, which can only be applied to unipartite networks. The local clustering coefficient is defined as the probability that two randomly chosen neighbors of a node *v* are connected. Global clustering coefficient is defined as the probability that two incident edges are completed by a third edge to form a triangle [95]. It can be expressed as [96]

29 $$\begin{aligned} CC = \frac{|\{u, v, w \in V| u \sim v \sim w \sim u\}|}{|\{u, v, w \in V| u \sim v {\ne } w \sim u\}|}, \end{aligned}$$

where $\sim$ means there is a connection between two nodes, and $\ne$ means node *v* and *w* are not the same one. The value of *CC* is between 0 and 1. A larger CC indicates that there are more triangles in the network and the greater the aggregation degree of nodes.

$\bullet$ **Assortativity Coefficient **($\hbox {AC}^1$) Assortativity is used to observe whether nodes with similar degrees tend to connect to each other. Assortativity coefficient is a Pearson correlation coefficient based on degree. Newman et al [97] propose the correlation function as

30 $$\begin{aligned} AC^1 = \frac{\sum _{j,k}^{}jk(e_{jk}-q_jq_k)}{\sigma _q^2}, \end{aligned}$$

where $q_k$ is the normalized distribution of the remaining degree, and is computed as

31 $$\begin{aligned} q_k = \frac{(k+1)p_{k+1}}{\sum _{j}^{}jp_j}, \end{aligned}$$

and $\sigma _q^2$ is a variance of the distribution of $q_k$, computed as

32 $$\begin{aligned} \sigma _q^2 = \sum _{k}^{}k^2q_k-\big [\sum _{k}^{}kq_k\big ]^2. \end{aligned}$$

Choosing an edge randomly, $e_{jk}$ is the joint probability that the degrees of the two endpoints are *j* and *k*, respectively. In general, $AD^1$ is between -1 and 1. A positive $AD^1$ indicates that the network has good assortativity, and a negative $AD^1$ reveals that the network is negatively correlated.

$\bullet$ **Power Law Exponent (PLE)** A network follows power law if its degree distribution follows

33 $$\begin{aligned} p(x) = Cx^{-\alpha }, \end{aligned}$$

where the constant $\alpha$ is the power law exponent [98]. If $\alpha$ is fixed, *C* is determined by the requirement that the sum of *p*(*x*) is 1. Complex networks obeyed power law distribution are referred as scale-free networks. A greater $\alpha$ implies a weaker scale-free network. Given a network, there are multiple ways to estimate $\alpha$. A robust method [99] calculates $\alpha$ as

34 $$\begin{aligned} \alpha = 1 + n(\sum _{v \in V}^{}\ln \frac{d(v)}{d_{min}})^{-1}. \end{aligned}$$

**Table 3:** Properties of complex networks (datasets used in experiments)

Category	Datasets	|*V*|	|*E*|	AD	CC	$AC^1$	PLE	EDE	$AC^2$
Coauthorship	APH [100]	18,771	198,050	22.0044	**0.6328**	**0.2013**	1.4245	0.9340	0.0272
	CM [100]	23,133	93,439	8.5462	**0.6417**	**0.1253**	1.5908	0.9525	0.0459
	GQ [100]	5241	14,484	6.4560	**0.5569**	**0.6392**	1.7423	0.9341	0.0353
	HPH [100]	12,006	118,489	20.9959	**0.6216**	**0.6295**	1.5142	0.8788	0.0355
	HTH [100]	9875	25,973	5.7435	**0.4816**	**0.2389**	1.7642	0.9512	0.0244
Computer	CAD [100]	26,475	53,381	4.0326	0.2082	–0.1946	**2.5086**	0.8381	0.0204
	GNT [101]	62,586	147,892	4.7275	0.0055	–0.0927	**2.0625**	0.9485	–
	RT [100]	6474	12,572	3.8838	0.2522	–0.1818	**2.4616**	0.8396	0.0880
Infrastructure	CHO [102]	1467	1298	1.9976	0.0000	–0.7248	4.7986	0.9124	0.0031
	EUR [103]	1174	1417	2.5120	0.0189	0.0900	2.2170	0.9854	0.0012
	OFS [104]	2939	15,677	10.7711	0.4555	0.0489	1.7168	0.8719	0.0408
	PG [95]	4941	6594	2.6691	0.0801	0.0035	2.2468	0.9783	0.0008
	USA [105]	1574	17,215	21.9008	0.5048	–0.1134	1.5462	0.8486	0.2180
Interaction Involving People	CHS [106]	7301	55,899	15.6793	0.1794	0.3705	1.4959	0.9248	0.0696
	CRE [107]	829	1473	3.5537	0.0058	–0.1645	2.0134	0.9584	0.2364
	UCI [108]	899	7019	15.6151	0.0705	–0.0945	1.4763	0.9256	0.3739
Protein–Protein Interaction	FGS [109]	2239	6432	5.7898	0.0403	–0.3318	2.0840	0.8550	0.1025
	STL [110]	1702	3155	3.8464	0.0063	–0.2020	2.2983	0.9007	0.0257
	VDL [111]	3023	6149	4.3169	0.0715	–0.1366	2.0531	0.9235	0.0698
	YST [112]	1846	2203	2.6722	0.0708	–0.2095	2.6030	0.9398	0.0213
Offline Social	ADE [113]	2539	10,455	**8.2355**	0.1467	0.2513	1.5141	**0.9824**	**0.2997**
	IFT [114]	410	2765	**13.4878**	0.4558	0.2258	1.4241	**0.9677**	**0.1945**
	JAZ [115]	198	2742	**27.6970**	0.6175	0.0202	1.3293	**0.9615**	**0.5720**
	PHY [116]	217	1839	**16.9493**	0.3628	0.0960	1.4928	**0.9815**	**1.7137**
	RSD [117]	241	923	**7.9487**	0.2192	–0.0842	1.7995	**0.9748**	**1.4240**
Online Social	AVG [118]	5155	39,285	15.5601	0.2527	–0.0957	1.5627	0.8926	0.1206
	BK [119]	58,228	214,078	7.5061	0.1734	0.0096	1.8880	0.9027	–
	DNC [120]	906	10,429	24.4617	0.5072	–0.1331	1.5529	0.8549	0.1405
	DB [121]	154,908	327,162	4.2240	0.0161	–0.1803	2.9706	0.8897	–
	EPN [122]	75,879	405,740	10.6947	0.1378	–0.0406	2.0258	0.8471	–
	FB [123]	2888	2981	2.0644	0.0272	–0.6682	25.5893	0.7087	0.0024
	G+ [123]	23628	39,194	3.3187	0.1742	–0.3887	3.9819	0.7677	0.0114
	GWL [119]	196,591	950,327	9.6681	0.2367	–0.0293	1.7307	0.9043	–
	HSS [124]	2426	16,630	16.0970	0.5401	0.0227	1.4541	0.9281	0.1029
	LMC [121]	104,103	2,193,083	42.1329	0.0544	–0.1468	1.3828	0.9003	–
	PRT [125]	10,680	24,316	4.5536	0.2659	0.2382	2.1092	0.9219	0.0112

The first column of the table is the network category, and the second one is a more specific classification. The properties measured from left to right are: number of nodes, number of edges, Average Degree, Clustering Coefficent, Assortativity Coefficient, Power Law Exponent, Edge Distribution Entropy, and Algebraic Connectivity. In each column, high values are highlighted in bold and low values are indicated by an underscore. High and low values are compared for categories by taking their mean values

$\bullet$ **Edge Distribution Entropy (EDE)** Entropy is used to measure the randomness of a system. Particularly, for a network, edge distribution entropy is computed as

35 $$\begin{aligned} EDE = \frac{1}{\ln n}\sum _{v \in V}^{} -\frac{d(v)}{2m}\ln \frac{d(v)}{2m}. \end{aligned}$$

It equals to one if all nodes have the same degree and is close to zero when all edges connect to a single node [126].

$\bullet$ **Algebraic Connectivity **($\hbox {AC}^2$) The algebraic connectivity is the second-smallest eigenvalue of the Laplacian matrix of a graph [127]. This measurement is greater than zero if and only if the graph is connected. Since the real networks do not always meet this condition, we consider the *Largest Connected Component* (LCC) instead of the entire network. It is used to analyze the robustness and the synchronizability of a network [128]. A higher algebraic connectivity suggests a better network connectivity.

### Datasets

In this section, we introduce the thirty-six datasets we used in experiments and divide them into seven types of complex networks according to their natural meaning. We also show the features of each type of networks we find from mining the datasets. Based on the statistical information in Table 3, the key characteristics of each type of complex networks are extracted, which lays an important foundation for the analysis of experimental results in Sect. 4.

$\bullet$ **Coauthorship Networks** In coauthorship networks [100, 129], nodes stand for a set of authors who have written papers together, and edges represent their collaboration relationships. AstroPh (APH) [100] is in the field of Astro Physics. CondMat(CM) [100] describes the collaborations of papers submitted to Condense Matter. GrQc (GQ) [100] is a coauthorship network of General Relativity and Quantum Cosmology. HepPh (HPH) [100] and HepTh (HTH) [100] show the collaborations between authors related to High Energy Physics and its theory category, respectively.

Higher clustering coefficients than most of the other networks imply that the small-world effect is significant in coauthorship networks. They have the highest and positive assortativity coefficient which shows their strong assortative. In other words, well-known authors tend to associate with each other.

$\bullet$ **Computer Networks** Due to the huge scales of computer networks, we conduct experiments on datasets named CAIDA (CAD) [100]: comes from a project that has the same name as the dataset; Route (RT) [100]: a communication network of autonomous systems collected from Route Views Project; and Gnutella (GNT) [101]. Nodes in computer networks are hosts or autonomous systems of the Internet. They exchange information through connections and form routing mechanisms.

According to the low power law exponent and the edge distribution entropy of computer networks, the edge distribution is skewed. In addition, negative assortativity coefficient shows that low-degree nodes prefer to connect with high-degree nodes.

$\bullet$ **Infrastructure Networks** An infrastructure network consists of physical engineering facilities that provide public services. Chicago (CHO) [102] shows the road transportation in the Chicago region, and Euroroad (EUD) [130] is an international E-road network. OpenFlights (OFS) [104] contains the information of flights collected by the OpenFlight project. PowerGrid (PG) [95] is an undirected network about the electrical grid of the Western US. USAir (USA) [105] shows a network of flights between US airports. These datasets compose the infrastructure networks used in the experiments.

Electric networks are similar to road networks. Their average degree is pretty low. The power law exponent and the edge distribution entropy are obviously higher than any other categories of networks, which indicates the edge distribution of this kind of network is more uniform. The connection between nodes only passes through a small number of local neighbors, resulting in a relatively small algebraic connectivity. Airline networks show different properties from them. Their average degrees are higher, and the edge distributions are more nonuniform which can be reflected by the power law exponents.

$\bullet$ **Interaction Networks Involving People** Most of the interaction networks involving people are bipartite networks that consist of people and items, where each edge represents an interaction [96]. For interaction networks, we use the following datasets : Chess (CHS) [106], Crime (CRE) [107] and UC Irvine (UCI) [108]. Chess is an anonymous dataset that represents the gaming relationships of chess players. Crime is a bipartite network, where nodes denote people or crimes. UC Irvine shows the forum messages posted by the students in the University of California, Irvine.

The degree distributions and the average degrees of interaction networks do not show a distinctive feature as the three networks mentioned earlier. There is no particularly distinctive features about this type of network.

$\bullet$ **Protein–Protein Interaction Networks** This kind of networks can be represented by a graph, where nodes and edges represent proteins and the interactions between them, respectively [131]. Figeys (FGS) [109], Stelzl (STL) [110], and Vidal (VDL) [111] are three PPI networks focusing on homo sapiens. Yeast (YST) [112] is a network of protein interactions in yeast.

We can draw a conclusion from Table 3 that the relationships between the proteins are sparse, and the probability that two proteins have no interaction even though they both interact with a third protein, is high. The assortativity coefficients are negative for four PPI networks, which implies that the molecules with high degrees tend to associate with low degrees.

$\bullet$ **Offline Social Networks** Offline social networks reflect the actual contacts between people, such as talking to each other, participating in activities together, or being physically close. The face-to-face interactions of people participating in big events, and the collaborations of musicians are typical offline social networks. Adole (ADE) [113] captures the connections between students in 1994/1995, and Infectious (IFT) [114] describes the face-to-face behaviors of visitors in the Infectious exhibition. Jazz (JAZ) [115] is a network that shows the collaborations between the Jazz musicians who have played in a band. Physicians (PHY) [116] is a directed network of physicians who are friends or interested in a discussion. Residence (RSD) [117] is a friendship network between the residents living in a residence hall located at an Australian university campus.

Statistics show that most of offline social networks are highly assortative which means people are more likely to associate with people of their own rank in real life. In addition, it is worth noting that the offline social networks have extremely strong scale-free characteristics and high edge distribution entropy which indicates a uniform degree distribution. The high average degrees and clustering coefficients indicate that the central network has obvious hierarchical characteristics. High algebraic connectivities means that all networks are well connected.

$\bullet$ **Online Social Networks** Online social networks consist of individuals and their connections in online social networking platforms and email systems. Plenty of platforms have become increasingly popular, such as Facebook, Twitter and YouTube [16]. Advogato (AVG) [118] is the trust network of an online community platform for the software developers. Brightkite (BK) [119] contains the friendship relations from a location-based social network. The network of Douban(DB) [121] comes from a Chinese online recommendation site. The data of DNC (DNC) [120] are generated from the Democratic National Committee email leak. Epinions (EPN) [122] is the trust network from the online social network Epinions. Facebook(FB) [123] consists of the friend lists. Each list comes from the survey participants using Facebook app. Google+ (G+) [123] is a network of Google+ user-user links. Gowalla (GWL) [119] is the friendship network of a namesake website. Hamsterster (HSS) [124] contains the contacts between users of the website Hamsterster. Livemocha (LMC) [121] is the network of an online language learning community. Pretty (PRT) [125] represents the interactions of people who use the Pretty Good Privacy algorithm.

Different from offline social networks, the assortativity coefficients of most networks are negative. It means that online networks break down invisible barriers between social classes, and the virtual relationships formed in social networks make it easier for ordinary people to connect with celebrities.

### Resources

This subsection summarizes valuable resources for investigating complex networks, including network datasets and network visualization tools.

#### Collections of Network Data

**SNAP **[132]. A collection of more than 50 large network datasets from tens of thousands of nodes and edges to tens of millions of nodes and edges, including social networks, web graphs, road networks, internet networks, citation networks, collaboration networks, and communication networks. **KONECT **[133]. The KONECT project has 1,326 network datasets in 24 categories. They have computed 56,300 graph statistics and generated 92,074 plots. AMiner Dateset [134]. The site offers datasets on COVID-19, scientific collaboration networks, multi-relationship networks, dynamic social networks, and many more related to machine learning and knowledge graph. Datasets Released for Reproducibility [135]. The website organized by the comunelab group provides a large number of multi-relational networks of varying degrees of complexity, including social networks and biological networks. Pajek datasets [136]. Many datasets in the early research of complex networks are derived from this collection. Network Repository [137]. The first interactive data and network data repository with real-time visual analytics. Network repository is not only the first interactive repository, but also the largest network repository with thousands of donations in 30+ domains (from biological to social network data). The Internet Topology Zoo [138]. This is an ongoing project to collect data network topologies from around the world. It currently has over two hundred and fifty networks in the Zoo, in a variety of graph formats for statistical analysis, plotting, or other network research.

#### Tools of Network Data

**Fig. 3:**
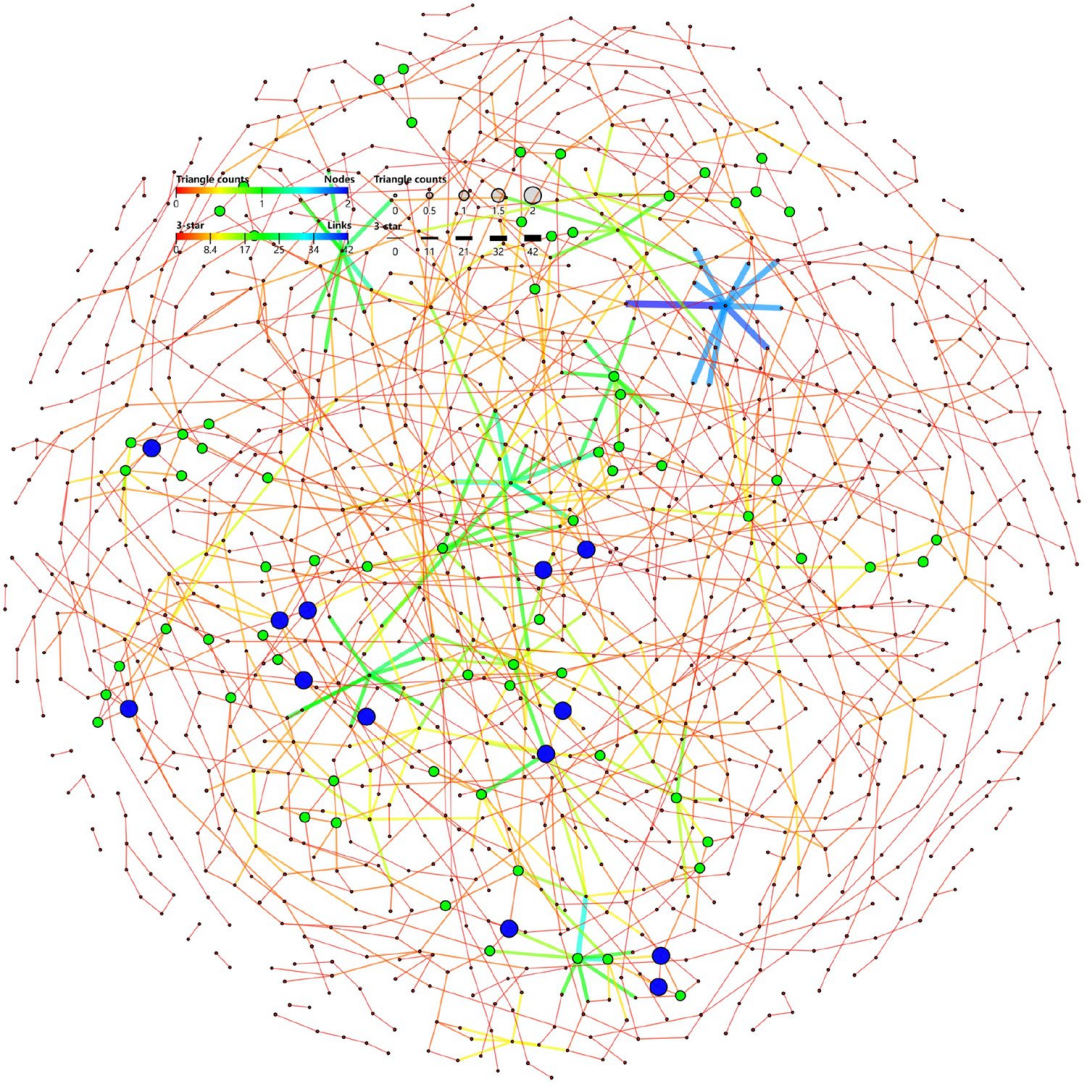
EUR network visualization using GraphVis

The research of complex network is inseparable from the statistics, calculation and drawing of various real or simulated networks. For general work, it can be done with software such as Pajek, Netdraw and Ucinet. Figure 3 is an example of visualizing the EUR network using GraphVis [137]. However, in some special scenarios, such as new models developed by oneself, corresponding modeling or calculation needs to be performed through programming. These two types of tools are summarized below.

**NetworkX **[139]. This is a Python package for the creation, manipulation, and study of the structure, dynamics, and functions of complex networks. **igraph ** [140].It is a collection of network analysis tools with the emphasis on efficiency, portability and ease of use. It can be programmed in R, Python, Mathematica and C/C++. **statnet ** [141]. statnet is a suite of R packages for the management, exploration, statistical analysis, simulation and vizualization of network data.

**Gephi **[142]. Gephi is a tool for data analysts and scientists keen to explore and understand all kinds of graphs and networks. **GraphVis ** [137]. GraphVis is a platform for interactive visual graph mining and relational learning. **MuxViz ** [143]. The platform for visualization and analysis of interconnected multilayer networks. It can be used as a library for the implementation of custom analysis or through an interactive browser-based graphical user interface to provide access to many customizable graphic options to render multilayer networks.

## Experiments and Analysis

In this section, we evaluate the methods mentioned in Sect. 2 on datasets of seven types of complex networks described in Sect. 3.2. The evaluation results^1^ will then be presented along with analysis which combine the properties of complex networks in a groundbreaking way.

### Evaluation Metrics

There are many evaluation methods of link prediction technology. In this paper, AUC [144], MRR [145] and HR@K are considered to evaluate the link prediction methods which measure results from different perspectives. AUC measures the quality of the method from the overall level. MRR focuses on the ranking of the edges which . HR@K considers the probability of existence of the edges in the first K position.

**Area Under the ROC Curve (AUC) **[144] AUC is the most suitable and commonly used metric to assess link prediction methods. This is owing to the imbalance distribution of link prediction datasets whose existing edges are notably less than absent edges, while AUC is unaffected by the distribution of the classes. It is tested as following: select one edge randomly from the test set, and select a non-existent edge randomly. Then we compare the scores of the two edges. If the former is greater than the latter, we add 1 to $t_1$; If the two are equal, we add 1 to $t_2$. Finally, the number of comparison time is *t*, and AUC can be computed as:

36 $$\begin{aligned} AUC=\frac{t_1+0.5t_2}{t} \end{aligned}$$

**Mean Reciprocal Rank (MRR) **[145] It is usually used to measure searching algorithms. If the content to be searched matches the first result, the score adds 1. If the content to be searched matches the second result, the score adds 0.5. If the content to be searched matches the *n*th result, the score adds 1/*n*. if there is no matching result, the score adds 0.

**Hit Ratio@K (HR) **[146] HR is often used to calculate the recall rate of the recommendation system. In general, the larger the index, the better the recommendation system. It can be computed as:

37 $$\begin{aligned} HR@K=\frac{M_{result}}{N_{neighbors}} \end{aligned}$$

The denominator $M_{result}$ is the total number of neighbors of a given node in the verification set, and the molecular $N_{neighbors}$ is the number of neighbors belonging to a given node in the verification set in the first K prediction results.

### System Setup

To construct a training set and a testing set, all existing links are randomly divided in a 9:1 ratio. We use AUC, MRR and HR@K(K= 1, 5, 10) to evaluate the performance of different approaches. Each experiment is repeated five times, and we use the average as the final result.

Most hyperparameters are inherited from the original paper of each method. Considering the time complexity and the settings of previous works, we reasonably hand-picked different parameters as follows. The parameter $\beta$ in KI is set to 0.01 and 0.001. In LPI, $\delta$ is fixed to 0.001. The value of $\phi$ in GLHN is tested for 0.9, 0.95 and 0.99. For RWR, the damping factor $\alpha$ is set to 0.85 and 0.95. LRW and SRW with $\alpha$ set to 0.85 are tested for the step length in 3, 4 and 5. The distance *r* is set to 5 in SR. MF is implemented by libFM, where the number of latent factors is fixed to $k = 5$. For DeepWalk, Node2vec, LINE and other network embedding methods, we end up with 128-dimensional embeddings and calculate the cosine distance of two nodes’ embedding as a link’s embedding. Paremeter tuning itself is a complex process. Since we would like to provide a quick and easy method which works for different types of networks, we only provide preliminary results where the parameters are set according to their original papers. Further adjustment may needed during one’s actual practice. All methods are implemented in Matlab and Python.

**Table 4:** Statistics of methods appearing in the top ten of the results

Methods/rank	1st	2nd	3rd	Top5	Top10	AvgRank
Common neighbor based	0	0.1	0	0.3	2.1	24
Path based	0	0.4	0.4	3.1	10.2	17.8
SBM	0	0	2	4	12	12.9
Classifier based	0	0	0.4	1	3.3	26.7
MF	1	6	3	17	22	6.8
DeepWalk	0	0	0	0	2	30.8
Node2vec	1	7	4	15	21	10.7
Struc2vec	1	4	5	18	25	7.1
GCN	0	0	0	0	0	35.8
GraphSAGE	0	0	1	1	2	26.1
WLNM	0	1	0	1	7	20.8
DGCNN	0	1	1	5	14	14.6
SEAL	0	6	3	17	25	5.9
LINE	0	0	1	2	4	25.9
SDNE	0	0	6	10	15	16.3
VERSE	28	1	1	30	31	1.3

### Results and Analysis

For the methods with multiple tested parameters, the best results are selected to report. Due to the limitations of space and time, we only evaluate a portion of the methods over large datasets of online social networks. The AUC values and the best methods are reported for each category of complex networks in all datasets. Some methods ran longer than 24 hours on some datasets, thus we terminate those methods, and represent the results as “-”. Surprisingly, some ground-breaking conclusions could be drawn from the empirical results. Due to space constraints, we show the AUC results of all methods on all kinds of networks, and combine the analysis of the attributes of the datasets in Sect. 3 to analyze the results in detail. MRR and HR are omitted for clarity and the complete results are publicly available at [147] for reference. We first analyze various methods and show their efficiency from an overall perspective, and then analyze particular performances of link prediction methods on different types of networks.

#### Overall Effects

Table 4 shows the number of times each approach ranks first, second, third, top five and top ten on all networks except for big datasets of online social networks. As we concentrate on network embedding-based methods, we show the average rank of other types of methods as a comparison.

It can be clearly seen from the Table 4 that the methods based on network embedding have the best performance, and their performance are less affected by network attributes which is due to its excellent ability to preserve network information. Examples of these excellent methods are VERSE, SEAL and Struc2vec, which capture network topology information very well and were developed specifically for link prediction task. It is worth noting that VERSE has outstanding performance and ranks first on almost every dataset. It rebuilds the similarity distribution between nodes by training a simple but expressive single-layer neural network, which is very effective in terms of accuracy and time efficiency. MF and Node2vec rely less on network properties and therefore perform well as well. Degree distribution does not affect the efficiency of the method based on matrix factorization. Since shallow encoders optimize an unique embedding vector for each node individually, the shortcoming of shallow network embedding methods comes from that no parameters are shared between nodes, and this will cause a sharp increase in parameter numbers and low computational efficiency. Different from shallow embedding methods, graph neural network-based methods use node features or local graph structures around each node as input to generate embeddings. Different graph neural network methods have different node representation capabilities, resulting in different performances.

As a summary, methods based on common neighbors are quite effective link prediction methods and are suitable for large-scale networks. When compared with advanced network embedding-based methods, they still have competitive performances on networks with high aggregation coefficients. However, due to the limited amount of information such methods preserve, the prediction accuracy of common neighbor-based methods is slightly lower than those of global indicators. Path-based methods also have mediocre performance, while ACT is not affected since it is based on the multiple-route distance diminishment. Path-based approaches take more information into consideration than common neighbor-based approaches, and the former can capture more global structures. The prediction results of probabilistic and statistical models are quite good, due to the reason that some additional information about network structures can be obtained by sampling the fitting and configuration of parameters. On the other hand, a key disadvantage is that the computation is extremely complex, thus it cannot be used to handle large-scale networks at present. Networks with higher aggregation coefficients are found to have a modular structure, so SBM performs better on this type of network. The performance of the methods based on classifier are generally poor, possibly owing to the imbalance of categories. Unlike similarity or probabilistic and statistical models-based methods which rank possible links based on the similarity between nodes or probability of link formation, the predicted number of links in each category cannot be well controlled.

**Table 5:** Runtime results(s)

	GQ	RT	OFS	USA	CHS	CRE	UCI	ADE	IFT	JAZ	AVG	DNC
CN	124.21	194.54	67.28	39.34	821.95	1.95	9.22	33.79	1.29	0.52	389.44	9.65
Salton	124.79	195.04	83.55	35.99	825.22	1.98	7.47	37.28	1.34	0.48	399.78	9.50
JI	124.46	223.07	71.88	35.83	829.50	2.00	7.56	35.08	1.37	0.48	418.16	9.73
Sorens	126.25	208.23	70.62	35.96	829.19	1.99	7.65	35.43	1.37	0.48	392.20	9.83
HPI	128.06	229.47	62.81	37.14	828.63	2.01	7.33	34.99	1.40	0.49	400.10	9.98
HDI	125.88	198.49	62.94	34.43	829.92	2.00	7.29	35.00	1.37	0.48	381.07	9.90
LLHN	121.78	197.09	61.23	33.92	822.72	1.95	7.46	34.38	1.41	0.48	383.64	9.88
AA	121.43	202.08	62.69	34.06	822.55	1.96	7.18	34.71	1.40	0.48	427.55	9.83
RA	120.72	191.37	67.22	33.58	824.08	1.95	7.21	34.56	1.36	0.48	398.08	9.62
PA	152.58	229.16	86.64	35.71	1021.88	2.08	7.82	40.14	1.41	0.48	445.87	9.67
LNBCN	121.05	194.31	83.59	33.62	822.94	1.95	7.53	33.93	1.36	0.48	386.68	9.58
LNBAA	120.47	190.72	69.02	33.75	822.87	1.95	7.58	34.32	1.35	0.47	385.93	9.82
LNBRA	241.59	381.55	134.23	67.81	1648.95	3.89	14.91	68.65	2.71	0.95	778.96	19.38
TSCN	195.59	797.75	105.83	55.25	2508.02	2.17	12.21	85.97	1.60	0.52	1084.99	15.21
LPI	122.66	210.41	67.20	35.51	938.39	1.95	7.45	35.58	1.41	0.47	429.35	9.66
KI	147.31	228.50	76.01	38.98	1031.91	2.08	8.15	42.09	1.39	0.66	462.51	10.29
GLHN	157.12	259.91	73.36	36.36	1050.12	2.14	9.39	42.71	1.43	0.78	484.94	10.59
ACT	195.35	359.22	83.06	37.59	1209.19	1.75	9.01	47.57	1.37	0.52	560.75	10.28
RWR	154.52	233.36	73.64	46.23	1122.68	1.67	8.38	46.38	1.35	0.51	509.63	10.08
SR	173.20	408.23	82.65	38.71	1141.32	1.85	8.03	43.54	1.36	0.51	528.61	10.77
LRW	124.27	210.68	65.92	35.47	941.17	1.61	7.93	34.94	1.20	0.49	438.03	10.81
SRW	123.86	208.96	68.22	38.00	944.38	1.95	7.53	35.07	1.20	0.49	430.63	10.11
MFI	148.67	224.34	70.02	48.72	1063.05	2.07	7.68	40.62	1.21	0.48	548.67	9.74
SBM	2164.01	25672.40	3628.33	1670.13	6707.33	240.09	656.54	1229.14	155.72	93.99	4420.39	922.64
Struc2Vec	153.72	164.48	93.44	55.54	838.63	3.33	12.89	54.34	2.33	1.13	409.51	19.24
GraphSAGE	1079.19	847.21	1236.27	1319.22	4379.00	112.35	358.64	614.30	145.14	153.84	3647.11	527.61
WLNM	816.10	7789.45	2349.60	5858.12	–	72.06	544.47	517.47	210.10	264.18	9500.13	2907.10
DGCNN	694.41	525.44	787.62	791.45	26738.46	64.67	289.21	489.54	113.08	105.23	2197.41	413.38
LINE	151.33	160.43	91.98	53.96	824.54	3.02	12.95	53.76	2.29	1.11	407.72	18.93
VERSE	129.67	152.72	73.78	43.69	194.27	22.29	22.33	57.92	10.94	4.97	127.39	25.27

#### Efficiency Evaluation

Table 5 lists the run time results of representative methods on datasets with comparative significance. We can analyze the scalability of each method through this set of experiments. SBM is extremely sensitive to the number of edges, so it is not suitable for datasets with large number of edges. On the contrary, for DGCNN, the increase of the edge amount does not affect the running time much. WLNM is not sensitive to the number of vertices but is sensitive to the number of edges, while VERSE is completely opposite to WLNM. GraphSAGE is not sensitive to vertex number increasing, and thus is suitable for datasets with a large number of vertices. From the perspective of time consumption, the average run time of common neighbor-based methods is the shortest, among which TSCN is the longest. When there is a need to get an initial result very quickly and does not have a strict restriction on accuracy, a common neighbor-based approach is a good choice. The methods based on probabilistic and statistical models can extract the underlying structure and obtain additional information of the networks by fitting the parameters, while they are time-consuming and are not applicable to deal with large-scale networks. Network embedding-based methods can achieve superior results, while the time consumption is acceptable meanwhile. Methods based on graph neural network run a little longer than other network embedding methods, while it could capture more abundant network information.

**Table 6:** AUC results of coauthorship networks, computer networks and infrastructure networks

	Coauthorship Networks					Computer Networks			Infrastructure Networks				
Methods	APH	CM	GQ	HPH	HTH	CAD	GNT	RT	CHO	EUR	OFS	PG	USA
CN	0.9854	0.9647	0.9145	0.9799	0.9000	0.9599	0.6887	0.7040	0.4980	0.5137	0.9364	0.5951	0.9534
Salton	0.9863	0.9651	0.9165	0.9796	0.9013	0.9602	0.6702	0.6827	0.4980	0.5134	0.9364	0.5893	0.9404
JI	0.9856	0.9646	0.9161	0.9797	0.9025	0.9699	0.6715	0.6821	0.4980	0.5132	0.9346	0.5968	0.9377
Sorens	0.9855	0.9652	0.9164	0.9796	0.9002	0.9601	0.6719	0.6835	0.4981	0.5153	0.9383	0.5956	0.9359
HPI	0.9861	0.9649	0.9181	0.9795	0.8993	0.9603	0.6846	0.6923	0.4976	0.5134	0.9346	0.5954	0.9079
HDI	0.9861	0.9651	0.9184	0.9796	0.9042	0.9598	0.6728	0.6836	0.4977	0.5141	0.9339	0.5914	0.9367
LLHN	0.9858	0.9648	0.9166	0.9793	0.9023	0.9596	0.6683	0.6805	0.4980	0.5134	0.9275	0.5949	0.8449
AA	0.9863	0.9653	0.9215	0.9794	0.9055	0.9602	0.6923	0.7072	0.4975	0.5121	0.9407	0.5895	0.9570
RA	0.9857	0.9654	0.9161	0.9798	0.9017	0.9604	0.6920	0.7019	0.4980	0.5141	0.9462	0.5966	0.9615
PA	0.9463	0.9126	0.7428	0.9561	0.7273	0.9124	0.8316	0.7467	0.2205	0.3036	0.8640	0.4540	0.9298
LNBCN	0.9862	0.9651	0.9194	0.9782	0.9025	0.9602	0.6927	0.7011	0.4979	0.5138	0.9418	0.5954	0.9547
LNBAA	0.9863	0.9653	0.9167	0.9800	0.9023	0.9604	0.6921	0.7030	0.4979	0.5135	0.9431	0.5934	0.9568
LNBRA	0.9865	0.9650	0.9178	0.9808	0.9062	0.9601	0.6923	0.7084	0.4980	0.5139	0.9415	0.5911	0.9617
TSCN	0.5438	0.7392	0.6369	0.5138	0.7562	0.5129	0.5102	0.5291	0.4720	0.6459	0.4944	0.6452	0.4390
LPI	0.9893	0.9716	0.9329	0.9841	0.9233	0.9694	0.7879	0.7624	0.4945	0.5371	0.9465	0.6482	0.9545
KI	0.9905	0.9757	0.9166	0.9849	0.8972	0.9731	0.7988	0.6939	0.4565	0.6451	0.9308	0.6602	0.1754
GLHN	0.9907	0.9793	0.8914	0.9862	0.8748	0.9757	0.7147	0.4394	0.7130	0.6128	0.8584	0.6492	0.6913
ACT	0.9485	0.9384	0.8103	0.9711	0.7764	0.9362	0.9359	0.8279	0.9847	0.9075	0.8896	0.9540	0.9304
RWR	0.9914	0.9761	0.9161	0.9853	0.8966	0.8481	0.8396	0.7798	0.4551	0.6594	0.9334	0.6697	0.9514
SR	0.9912	0.9759	0.9188	0.9851	0.8945	0.7816	0.7481	0.5942	0.4716	0.7021	0.9159	0.7689	0.8507
LRW	0.9913	0.9757	0.9318	0.9857	0.9215	0.8466	0.8338	0.7833	0.4950	0.5401	0.9545	0.6522	0.9616
SRW	0.9915	0.9755	0.9302	0.9859	0.9229	0.8427	0.8310	0.7813	0.4932	0.5392	0.9504	0.6521	0.9604
MFI	0.9902	0.9748	0.9103	0.9842	0.8977	0.8286	0.8022	0.7054	0.4536	0.6472	0.9237	0.6650	0.9236
SBM	–	–	0.8939	–	0.8517	–	0.9375	0.9391	0.8835	0.6823	0.9373	0.6534	0.9688
SVM	0.7324	0.8346	0.8619	0.7726	0.8845	0.7295	0.8197	0.7864	0.8097	0.6111	0.8063	0.5002	0.7152
KNN	0.8391	0.8980	0.9043	0.7818	0.9027	0.6302	0.6244	0.5549	0.5846	0.5485	0.7847	0.5970	0.7153
DT	0.5972	0.5998	0.5883	0.6053	0.5869	0.5588	0.5708	0.5233	0.5386	0.5812	0.5135	0.5494	0.5917
Bayes	0.9667	0.9611	0.9394	0.9268	0.9277	0.9110	0.9056	0.7967	0.5248	0.5030	0.8761	0.7629	0.7966
LR	0.7584	0.6853	0.6879	0.8791	0.5618	0.9191	0.8602	0.7861	0.4157	0.3385	0.6842	0.4572	0.7430
MLP	0.7233	0.7659	0.7768	0.6305	0.7308	0.6857	0.8034	0.7655	0.4209	0.3426	0.6807	0.4634	0.7451
MF	–	–	0.9188	0.9628	0.9206	0.9782	0.9745	–	0.9699	0.8416	0.9619	0.8942	0.9702
DeepWalk	0.6935	0.7250	0.7753	0.6829	0.6718	0.5919	0.5784	0.6702	0.8873	0.7848	0.6896	0.7251	0.6021
Node2vec	0.9925	0.9701	0.9349	0.9407	0.9264	0.9722	0.9518	0.9617	0.9889	0.9805	0.9588	0.9904	0.8544
Struc2vec	0.9931	0.9832	0.9458	0.9497	0.9399	0.9835	0.9597	0.9739	0.8671	0.8973	0.9598	0.8257	0.9235
GCN	0.6372	0.5730	0.5685	0.5587	0.5960	0.6410	0.5900	0.6896	0.4970	0.5338	0.5962	0.5144	0.5356
GraphSAGE	0.8002	0.7508	0.7730	0.8939	0.7323	0.8356	0.7923	0.7988	0.6743	0.6515	0.8536	0.6851	0.8758
WLNM	0.8563	0.8819	0.9269	–	0.8821	0.7268	0.7441	0.8802	0.9149	0.7042	0.9319	0.7701	0.9088
DGCNN	0.9169	0.9161	0.9100	0.9168	0.8404	0.8632	0.9232	0.9108	0.9789	0.9667	0.9406	0.9259	0.9311
SEAL	0.9925	0.9913	0.9793	0.9902	0.9659	–	0.9587	–	0.9705	0.7796	0.9730	0.7864	0.9600
LINE	0.9261	0.9691	0.9615	0.9598	0.9366	0.5659	0.5484	0.5285	0.4253	0.5957	0.8275	0.7311	0.4646
SDNE	0.8814	0.8625	0.7518	0.8325	0.8172	0.9609	0.9498	0.9503	0.9655	0.9695	0.9253	0.9648	0.9054
VERSE	0.9992	0.9994	0.9979	0.9992	0.9978	0.9977	0.9853	0.9965	0.9655	0.9863	0.9956	0.9948	0.9928

#### Particular Performances on Different Types of Networks

The AUC results of coauthorship networks, computer networks and infrastructure networks are reported in Table 6. Table 7 shows the results on interaction networks involving people, protein–protein interaction networks and offline social networks. Tables 8 and 9 show the outcomes of small and large datasets of online social networks, respectively. We will analyze particular performances of some methods on different types of networks, where the inconsistency comes from different characteristics of different kinds of networks. Methods that are not affected by network attributes are mentioned in Sect. 4.3.1 and will not be repeated here. For example, VERSE performs well on any kind of networks and will not be discussed in this section.

**Table 7:** AUC results of interaction networks involving people, protein–protein interaction networks and offline social networks

	Interaction networks involving people			Protein–protein interaction networks				Offline social networks				
Methods	CHS	CRE	UCI	FGS	STL	VDL	YST	ADE	IFT	JAZ	PHY	RSD
CN	0.8783	0.5043	0.7262	0.5526	0.5238	0.6162	0.5917	0.7652	0.9362	0.9576	0.9085	0.8407
Salton	0.8784	0.5051	0.6923	0.5474	0.5229	0.6169	0.5894	0.7695	0.9367	0.9659	0.9208	0.8394
JI	0.8779	0.5045	0.6998	0.5486	0.5253	0.6153	0.5881	0.7700	0.9401	0.9580	0.9212	0.8365
Sorens	0.8828	0.5043	0.6905	0.5485	0.5249	0.6146	0.5897	0.7678	0.9369	0.9615	0.9156	0.8376
HPI	0.8747	0.5036	0.6924	0.5506	0.5248	0.6179	0.5904	0.7647	0.9321	0.9484	0.9166	0.8389
HDI	0.8796	0.5057	0.6945	0.5479	0.5242	0.6179	0.5899	0.7712	0.9358	0.9487	0.9135	0.8333
LLHN	0.8740	0.5052	0.6643	0.5473	0.5252	0.6157	0.5894	0.7710	0.9246	0.9031	0.8955	0.8357
AA	0.8814	0.5054	0.7316	0.5561	0.5272	0.6176	0.5878	0.7726	0.9352	0.9611	0.9179	0.8399
RA	0.8787	0.5061	0.7269	0.5537	0.5246	0.6176	0.5920	0.7661	0.9368	0.9694	0.9207	0.8437
PA	0.9316	0.6189	0.8369	0.8080	0.6890	0.7463	0.4885	0.6147	0.7134	0.7671	0.6212	0.5200
LNBCN	0.8787	0.5056	0.7345	0.5783	0.5278	0.6138	0.5890	0.7699	0.9327	0.9594	0.9125	0.8407
LNBAA	0.8828	0.5060	0.7294	0.5742	0.5268	0.6170	0.5873	0.7701	0.9386	0.9636	0.9194	0.8470
LNBRA	0.8788	0.5040	0.7282	0.5765	0.5256	0.6148	0.5894	0.7748	0.9356	0.9726	0.9113	0.8368
TSCN	0.5362	0.5507	0.5231	0.4760	0.5737	0.5144	0.5747	0.6996	0.5365	0.5141	0.6266	0.8891
LPI	0.9391	0.5876	0.8016	0.8016	0.7156	0.7667	0.6178	0.8430	0.9526	0.9509	0.9055	0.9062
KI	0.9311	0.5770	0.8087	0.7550	0.6546	0.7507	0.5742	0.8844	0.9525	0.9425	0.9039	0.9164
GLHN	0.9099	0.5022	0.5586	0.4939	0.5635	0.6513	0.5031	0.8786	0.9362	0.8082	0.8379	0.9062
ACT	0.8555	0.7001	0.8349	0.8982	0.8421	0.8162	0.7681	0.6570	0.8088	0.7773	0.6581	0.5290
RWR	0.9468	0.5870	0.8037	0.8031	0.6777	0.7727	0.5803	0.9047	0.9606	0.9500	0.9118	0.9194
SR	0.9398	0.4834	0.6586	0.3261	0.5220	0.7006	0.5845	0.9022	0.9510	0.8991	0.9057	0.9193
LRW	0.9444	0.5881	0.8151	0.8494	0.7220	0.7684	0.6184	0.8471	0.9613	0.9529	0.9221	0.9149
SRW	0.9440	0.5893	0.7863	0.8353	0.7089	0.7665	0.6134	0.8433	0.9647	0.9663	0.9246	0.9163
MFI	0.9417	0.5538	0.7774	0.7453	0.6419	0.7347	0.5755	0.9041	0.9534	0.9222	0.9129	0.9121
SBM	0.9015	0.6562	0.8370	0.9532	0.8557	0.8206	0.7966	0.8360	0.9423	0.9172	0.8599	0.8891
SVM	0.7586	0.6429	0.7157	0.8864	0.7568	0.8125	0.6518	0.7316	0.7175	0.7023	0.7359	0.7618
KNN	0.7315	0.5824	0.6038	0.7316	0.5873	0.7519	0.5940	0.6657	0.7191	0.7524	0.7742	0.7369
DT	0.5200	0.5109	0.5347	0.5021	0.6195	0.5472	0.5268	0.5268	0.5481	0.5816	0.5364	0.5660
Bayes	0.8126	0.6318	0.6234	0.7964	0.7122	0.8195	0.7616	0.7586	0.7499	0.8978	0.9005	0.8993
LR	0.7557	0.6291	0.6809	0.8149	0.7233	0.7157	0.5763	0.6584	0.6815	0.9038	0.7125	0.7067
MLP	0.7250	0.6314	0.6922	0.8075	0.7354	0.7341	0.6008	0.6596	0.6971	0.7833	0.7654	0.8050
MF	0.9368	0.8925	0.8953	0.9657	0.8904	0.9026	0.9269	0.8925	0.9431	0.9784	0.9529	0.9211
DeepWalk	0.6259	0.6152	0.5264	0.5639	0.5967	0.6654	0.6872	0.6562	0.7254	0.6533	0.6194	0.8582
Node2vec	0.9436	0.9643	0.6366	0.8700	0.9416	0.9497	0.9765	0.9623	0.9378	0.9046	0.9297	0.9658
Struc2vec	0.9466	0.9713	0.8533	0.9488	0.8815	0.9108	0.9340	0.9737	0.9604	0.9325	0.9308	0.9655
GCN	0.6901	0.5475	0.6481	0.6140	0.5795	0.6054	0.5043	0.5153	0.5505	0.5967	0.5359	0.6056
GraphSAGE	0.7904	0.6012	0.7261	0.7339	0.6898	0.6624	0.6819	0.7066	0.8083	0.7958	0.7478	0.6740
WLNM	0.5406	0.5984	0.6994	0.8230	0.7369	0.7823	0.7506	0.7940	0.8457	0.8459	0.7677	0.7634
DGCNN	0.9139	0.8714	0.9361	0.9382	0.8399	0.8199	0.9114	0.8873	0.9406	0.9190	0.9108	0.9067
SEAL	0.9651	0.8127	0.8597	0.9593	0.9128	0.9017	0.9051	0.8961	0.9528	0.9539	0.8898	0.9124
LINE	0.8119	0.5690	0.5169	0.5214	0.5599	0.6960	0.7596	0.8274	0.9514	0.8838	0.8696	0.9023
SDNE	0.9312	0.9576	0.6153	0.8975	0.9217	0.9366	0.9518	0.9549	0.9421	0.9133	0.9152	0.9267
VERSE	0.9930	0.9687	0.9686	0.9915	0.9815	0.9897	0.9938	0.9909	0.9925	0.9701	0.9698	0.9714

**Coauthorship Networks** SEAL performs well on all tested datasets. LINE, Node2vec and Struc2vec follow closely behind, which reveals that network embedding methods are suitable for coauthorship networks. High clustering coefficients and average node degrees ensure that subgraphs of coauthorship networks preserve sufficient local information. Therefore, methods based on local information such as common neighbor-based methods perform well. For coauthorship networks, authors who belong to the same organization have a high probability of publishing papers together. However, there is a considerable portion of links between different organizations. Hence, path-based methods are also competitive. In a word, when meets time and space consumptions, SEAL is recommended for coauthorship networks with high clustering coefficients, assortativity coefficients, and strong scale-free features.

**Computer Networks** Computer networks present the properties of low average degree, weak connectivity and skewed degree distribution, resulting in the difficulty to predict links by obvious topology information. In view of this situation, Struc2vec performs surprisingly well on CAD, RT and shows competitive performance on GNT. Skewed degree distributions lead to apparent community structure properties, which contributes to the good performance of Struc2vec. In addition, MF, Node2vec and SDNE also achieve good performances. According to above analysis, network embedding methods with matrix factorization and random walk are recommended for computer networks.

**Infrastructure Networks** According to the network attributes shown in Table 3, the airlines network has a high average degree and a low power law exponent with a high clustering coefficient. In terms of the overall effects, DGCNN has the most competitive performance on this kind of networks. Node2vec and SDNE work well in electrical networks and roads networks, while have an average performance on airlines datasets. Because of the uniform degree distribution, low clustering coefficient and numerous low-degree nodes which make it difficult to capture local information well, common neighbor-based methods and most path-based methods show bad results in infrastructure networks. However, ACT is based on the multiple-route distance diminishment, which makes ACT will not be affected by those properties of infrastructure networks.

**Table 8:** AUC results of small datasets of online social networks

Methods/datasets	AVG	DNC	FB	G+	HSS	PTY
CN	0.9008	0.9667	0.5033	0.9549	0.9616	0.8466
Salton	0.8834	0.9319	0.5249	0.9628	0.9703	0.8462
JI	0.8812	0.9309	0.4837	0.9626	0.9694	0.8468
Sorens	0.8803	0.9317	0.5061	0.9627	0.9691	0.8471
HPI	0.8798	0.8256	0.4969	0.9625	0.9675	0.8470
HDI	0.8827	0.9267	0.5276	0.9626	0.9712	0.8466
LLHN	0.8707	0.8074	0.4739	0.9625	0.9627	0.8468
AA	0.9009	0.9717	0.4965	0.9627	0.9709	0.8472
RA	0.9076	0.9714	0.5142	0.9624	0.9746	0.8474
PA	0.8946	0.9325	0.4590	0.9112	0.9447	0.8453
LNBCN	0.9033	0.9681	0.4879	0.9629	0.9704	0.8471
LNBAA	0.9036	0.9695	0.5384	0.9627	0.9705	0.8471
LNBRA	0.9063	0.9716	0.5168	0.9628	0.9736	0.8472
TSCN	0.5061	0.5077	0.5485	0.5137	0.5394	0.5925
LPI	0.9296	0.9629	0.4861	0.9705	0.9827	0.8579
KI	0.9262	0.9515	0.4327	0.9754	0.9835	0.8503
GLHN	0.6796	0.6327	0.4692	0.9792	0.9826	0.8618
ACT	0.8969	0.9532	0.9875	0.9829	0.9466	0.9622
RWR	0.9290	0.9617	0.4526	0.7304	0.9843	0.8505
SR	0.8637	0.8190	0.4368	0.6765	0.9802	0.8534
LRW	0.9302	0.9668	0.4457	0.7658	0.9855	0.8641
SRW	0.9292	0.9660	0.5135	0.7653	0.9807	0.8638
MFI	0.9115	0.9519	0.4983	0.7222	0.9814	0.8507
SBM	0.9105	0.9810	0.9856	–	–	–
SVM	0.6523	0.8546	0.8367	0.9116	0.8382	0.7878
KNN	0.6849	0.8367	0.5543	0.6946	0.9017	0.7896
DT	0.5694	0.6318	0.5230	0.6353	0.6006	0.6253
Bayes	0.8368	0.8416	0.6644	0.9651	0.9485	0.9003
LR	0.6316	0.8564	0.5891	0.9799	0.8847	0.5842
MLP	0.5849	0.8648	0.4492	0.8836	0.7934	0.7197
MF	0.9628	0.9837	0.9918	–	0.9572	0.9156
DeepWalk	0.5533	0.6352	0.8029	0.5697	0.6984	0.7932
Node2vec	0.8869	0.7138	0.9821	0.9602	0.9526	0.9859
Struc2vec	0.9044	0.9703	0.9762	0.9674	0.9701	0.9537
GCN	0.5831	0.7647	0.5694	0.5981	0.5630	0.6079
GraphSAGE	0.7591	0.9162	0.9954	0.8835	0.7794	0.7988
WLNM	0.8823	0.9497	0.9987	0.7756	0.9269	0.9537
DGCNN	0.9439	0.9420	0.9066	0.9131	0.9605	0.9226
SEAL	0.9486	0.9763	0.9898	–	0.9833	0.9684
LINE	0.7040	0.6775	0.5861	0.4669	0.8998	0.9218
SDNE	0.8725	0.7076	0.9309	0.9536	0.9475	0.9802
VERSE	0.9910	0.9970	0.9997	0.9998	0.9978	0.9916

**Interaction Networks Involving People** Except for VERSE, there is no single method that performs well on all three datasets. This may be caused by inconsistent statistical properties. Methods based on network embedding with random walk are worth of attentions, especially Struc2vec, which performs better than other methods on the CRE dataset and shows competitive performance on other datasets as well. On the whole, DGCNN performs best among all the methods. Thus Struc2vec and DGCNN can be considered as a quick-pick method for this kind of networks. For bipartite datasets, there is no common neighbor between different roles, which makes it difficult to predict these links for the methods based on common neighbors.

**Protein–protein Interaction Networks** Although Node2vec obtains the most impressive results on three datasets, it has a mediocre performance on the FGS dataset. Considering the results of protein–protein interaction networks and infrastructure networks jointly, MF is also an excellent method when a network shows less connectivity and low cluster coefficient. LINE performs poor on the PPI networks because of the low cluster coefficient, while Struc2vec, SEAL, and SDNE have a better performance. To sum up, methods based on matrix factorization and random walk tend to be more suitable for link prediction tasks on PPI networks.

**Offline Social Networks** Methods for each dataset in Table 7 do not show a consistent trend. Uniform degree distribution, high assortativity and good connectivity indicate that the information of offline social networks are evenly distributed. Methods based on network embedding, especially random walk-based approaches generally work well on offline social networks. The reason may be that the coverage of random walk is wider and more comprehensive.

**Table 9:** AUC results of big datasets of online social networks

Methods/datasets	BK	DB	EPN	GWL	LMC
CN	0.8194	0.5978	0.8685	0.8837	0.7826
Salton	0.8565	0.6059	0.9131	0.8972	0.7634
JI	0.8216	0.5999	0.8607	0.8775	0.7781
Sorens	0.8217	0.5797	0.8609	0.8773	0.7795
HPI	0.8542	0.6214	0.9012	0.8901	0.7783
HDI	0.8186	0.5830	0.8620	0.8775	0.7774
LLHN	0.8569	0.6163	0.9088	0.8907	0.7653
AA	0.8205	0.5886	0.8709	0.8801	0.7829
RA	0.8197	0.5879	0.8716	0.8806	0.7812
PA	0.8312	0.6691	0.8901	0.8674	0.9215
LNBCN	0.8201	0.5877	0.8725	0.8761	0.7903
LNBAA	0.8193	0.5874	0.8707	0.8757	0.7899
LNBRA	0.6400	0.5876	0.8724	0.8760	0.7886
DeepWalk	0.6426	0.5437	0.6028	0.6514	0.5386
Node2vec	0.9731	0.9714	0.9269	0.9794	0.8239
Struc2vec	0.9783	0.9796	0.9298	0.9852	0.8315
GCN	0.6563	0.6047	0.7937	–	–
DGCNN	0.9276	0.9444	0.9520	0.9419	0.9298
LINE	0.8383	0.6234	0.6454	0.8098	0.6875
SDNE	0.9705	0.9685	0.9193	0.9718	0.8197
VERSE	0.9969	0.9970	0.9964	0.9980	0.9865

**Online Social Networks** Table 8 shows the AUC results of small online social networks. High average degree and cluster coefficient provide enough information for link prediction on online social networks. However, some path-based methods involve too much noise. Unbalanced degree distribution and strong scale-free lead to the fact that there is no single method performs well on all datasets. Although MF and SEAL do not obtain the best results in all datasets, their performance are generally very close to the best methods. As can be seen from the table, network embedding-based link prediction methods performs well on this kind of networks and are excellent methods to choose from.

For big online social networks, we show the results in Table 9. We only show a portion of the methods over large datasets of online social networks, while the rests cannot complete the task with time and space limitations. VERSE kept its usual dominant position. Other than that, DGCNN performs best among these networks since it can learn more expressive representations than others. Node2vec and Struc2vec perform better than other methods on all datasets. This is reasonable because Node2vec and Struc2vec capture more information than common neighbor-based methods, which is achieved at the cost of more time consumption. For common neighbor-based methods, PA, which only considers the degree of nodes, has surprisingly good performance in LMC. When PA appears as the best method, it is significantly better than other methods. It can be found that LMC has extremely high average degree, which compromises the performance of other common neighbor-based methods.

## Conclusions

In this survey, we have conducted, as far as we know, the most comprehensive experimental overview of the link prediction methods that have been proposed till now on complex networks. We propose a scientific taxonomy, which reasonably classifies the representative link prediction methods according to their internal principles. We then divide thirty-six datasets into seven different types of networks according to their natural meaning, and extract network property features for each type of the networks. Next, we analyze the properties of different type of networks in detail. Full-scale experiments have been performed for forty-two link prediction methods on above mentioned thirty-six datasets. On the basis of statistical analysis of the experimental results, we further analyze them in detail in order to reveal the methods with good performance and recommend appropriate link prediction methods for each type of networks. In addition, observing that the methods based on network embedding provide new solutions for the tasks of link prediction, while the complete investigation of such methods has been missing. One of the important contributions of this paper is to fill in the gap of the research of link prediction methods based on network embedding.

## Data Availability

https://github.com/whxhx/Link-Prediction-Methods.

## Footnotes

1 All datasets, codes and complete results are publicly available at https://github.com/whxhx/Link-Prediction-Methods.
